# Integrated mRNA-miRNA transcriptome analysis of bladder biopsies from patients with bladder pain syndrome identifies signaling alterations contributing to the disease pathogenesis

**DOI:** 10.1186/s12894-021-00934-0

**Published:** 2021-12-07

**Authors:** Ali Hashemi Gheinani, Akshay Akshay, Mustafa Besic, Annette Kuhn, Irene Keller, Rémy Bruggmann, Hubert Rehrauer, Rosalyn M. Adam, Fiona C. Burkhard, Katia Monastyrskaya

**Affiliations:** 1 Functional Urology Research Group, Department for BioMedical Research DBMR, University of Bern, Bern, Switzerland; 2 Urological Diseases Research Center, Boston Children’s Hospital, Boston, MA USA; 3 Department of Surgery, Harvard Medical School, Boston, MA USA; 4 Broad Institute of MIT and Harvard, Cambridge, MA USA; 5 Department of Gynaecology, Inselspital University Hospital, 3010 Bern, Switzerland; 6 Interfaculty Bioinformatics Unit, University of Bern, Bern, Switzerland; 7 Functional Genomics Center Zurich, ETH Zurich/University of Zurich, Zurich, Switzerland; 8 Department of Urology, Inselspital University Hospital, 3010 Bern, Switzerland

**Keywords:** Bladder, Pain, Cystitis, Gene, miRNA, Signaling pathway, Next-generation sequencing

## Abstract

**Background:**

Interstitial cystitis, or bladder pain syndrome (IC/BPS), is a chronic bladder disorder characterized by lower abdominal pain associated with the urinary bladder and accompanied by urinary frequency and urgency in the absence of identifiable causes. IC/PBS can be separated into the classic Hunner’s ulcerative type and the more prevalent non-ulcerative disease. Our aim was to unravel the biological processes and dysregulated cell signaling pathways leading to the bladder remodeling in non-ulcerative bladder pain syndrome (BPS) by studying the gene expression changes in the patients’ biopsies.

**Methods:**

We performed paired microRNA (miRNA) and mRNA expression profiling in the bladder biopsies of BPS patients with non-Hunner interstitial cystitis phenotype, using comprehensive Next-generation sequencing (NGS) and studied the activated pathways and altered biological processes based on the global gene expression changes. Paired mRNA-miRNA transcriptome analysis delineated the regulatory role of the dysregulated miRNAs by identifying their targets in the disease-induced pathways.

**Results:**

EIF2 Signaling and Regulation of eIF4 and p70S6K Signaling, activated in response to cellular stress, were among the most significantly regulated processes during BPS. Leukotriene Biosynthesis nociceptive pathway, important in inflammatory diseases and neuropathic pain, was also significantly activated. The biological processes identified using Gene Ontology over-representation analysis were clustered into six main functional groups: cell cycle regulation, chemotaxis of immune cells, muscle development, muscle contraction, remodeling of extracellular matrix and peripheral nervous system organization and development. Compared to the Hunner’s ulcerative type IC, activation of the immune pathways was modest in non-ulcerative BPS, limited to neutrophil chemotaxis and IFN-γ-mediated signaling. We identified 62 miRNAs, regulated and abundant in BPS and show that they target the mRNAs implicated in eIF2 signalling pathway.

**Conclusions:**

The bladders of non-ulcerative BPS patients recruited in this study had alterations consistent with a strong cell proliferative response and an up-regulation of smooth muscle contractility, while the contribution of inflammatory processes was modest. Pathway analysis of the integrated mRNA-miRNA NGS dataset pinpointed important regulatory miRNAs whose dysregulation might contribute to the pathogenesis. Observed molecular changes in the peripheral nervous system organization and development indicate the potential role of local bladder innervation in the pain perceived in this type of BPS.

**Supplementary Information:**

The online version contains supplementary material available at 10.1186/s12894-021-00934-0.

## Background

Interstitial cystitis, or bladder pain syndrome (IC/BPS), is a chronic bladder disorder characterized by lower abdominal pain associated with the urinary bladder and accompanied by day-time and/or night-time urinary frequency and urinary urgency, in the absence of identifiable causes such as bacterial infection [1]. BPS affects people of different age groups with a higher prevalence in women (45 cases per 100,000) compared to men (8 cases per 100,000) [2]. An estimated 2.70% to 6.53% (or 3.3 to 7.9 million) of US women 18 years old or older reported BPS/IC symptoms [3]. IC/PBS can be separated into two subtypes: classic Hunner’s ulcerative type, characterized by Hunner’s ulcers or haemorrhages in the bladder and the more prevalent non-ulcerative disease [4]. The aetiology of BPS is unknown, and its treatment is largely empiric. The main cause of IC/PBS is thought to be a persistent bladder inflammation, possibly brought about by the deficiency of the glycosaminoglycan covering the urothelium surface that results in leaky urothelium and subsequent activation of afferent nerves and neurogenic inflammation. Growing evidence suggests that there is a morphological and functional distinction between IC/BPS with Hunner lesions and IC/BPS without Hunner lesions: IC/BPS with Hunner lesions is an inflammatory disorder characterized by B cell abnormalities and epithelial denudation, while IC/BPS without Hunner lesions shows minimal histological changes [5].

Studying gene expression changes in disease might shed light on the underlying biological processes and help developing specific therapies. Previously, investigating the expression levels of genes implicated in epithelial permeability, bladder contractility, and inflammation, and the associated regulatory microRNAs in BPS patients’ bladder biopsies, we showed a significant down-regulation of tight junction proteins zona occludens-1, junctional adherins molecule-1, and occludin, indicative of increased urothelial permeability, and concomitant up-regulation of bradykinin B(1) receptor, cannabinoid receptor CB1 and muscarinic receptors M3-M5, pointing to the inflammation-driven smooth muscle contractility activation []. BPS induced an up-regulation of acid-sensing ion channel 2a and 3 mRNA in the bladder [6], while causing a decrease in the levels of anti-inflammatory annexin A1 in the urothelium [7].

Recent advances in Next-Generation Sequencing (NGS) allow comprehensive characterization of the gene expression profiles in different disease states, with subsequent pathway analysis revealing the involved pathogenetic mechanisms. To date, several transcriptome studies were carried out in human IC/BPS patients using gene expression microarrays in biopsies [8] and urine sediment [9]; QPCR panels of 96 inflammatory mediators [10], NGS in urine sediment [11] and bladder biopsies [12].

In order to investigate the pathogenetic changes and study the regulatory role of miRNAs in BPS, we performed an integrated analysis of miRNA and mRNA paired expression profiling in the bladder biopsies of BPS human patients with non-Hunner IC phenotype, previously characterized by other methodology [7, 13], using comprehensive Next-generation sequencing (NGS)-derived transcriptome data. We studied the activated pathways and altered biological processes based on the global gene expression changes and explored the presence and regulatory role of the individual miRNA targets in these pathways.

## Methods

### Patient selection

Patient recruitment and cold cup bladder dome biopsy collection from 14 controls and 22 BPS patients was described in our earlier published study [13]. Permission to conduct this study was obtained from the Ethics Committee of the Canton of Bern (KEK 146/05), and all subjects gave written informed consent. All subjects underwent a complete urological evaluation (including medical history, physical examination, urine culture, flexible urethrocystoscopy). In addition, all subjects with the BPS underwent uroflowmetry, post void residual (PVR) and multichannel urodynamic investigations including filling cystometry and pressure-flow studies. All patients had confirmed BPS without Hunner’s lesions. Patient selection and evaluation was described previously [13]. Full information on patients is given in the Additional file 1. Patients were divided in 2 groups:

1. Group 1: Control—asymptomatic patients undergoing cystoscopy for other reasons (e.g. stent placement for stone disease, microhematuria evaluation, fistula closure) (n = 6 for NGS, n = 8 for QPCR validation). All QPCR controls and 2 out of 4 NGS controls were female, average age was 51.8 ± 21 (QPCR) and 46.8 ± 6.7 (NGS).
2. Group 2: BPS—patients with pain (> 3 months) considered to be located in the bladder and/or frequency, urgency and nocturia (n = 6 for NGS, n = 16 for QPCR validation). All patients were female, average age 45.7 ± 15 (QPCR) and 38 ± 11 (NGS).

Biopsy samples from all subjects underwent a routine microscopic examination by a trained pathologist (H&E staining, staining for mast cell tryptase, staining for S100 proteins and PGP 9.5 for nerve fibres). No patient had an increased postvoid residual. Urodynamic studies showed a median cystometric bladder volume of 200 ml (range 60 – 1000), one patient had a high capacity bladder with a volume > 1000 ml and 2 patients had a normal capacity, all of the remaining ones had low capacity bladders. Histopathological evaluation showed chronic inflammation (lymphoplasmocytic infiltration, interstitial edema and/or hyperemia of the blood vessels with dilated lumina) in all but 4 patients, and 17 demonstrated increased mast cells (MC) in the smooth muscle (≥ 20 MC/mm^2^) (Additional file 1). Cold cup biopsies from the bladder dome, two biopsies per subject were collected and stored in RNAlater (Qiagen) at − 70 °C until RNA isolation. Total RNA was isolated from bladder dome biopsies using the miRVana miRNA isolation kit (Ambion) and the RNA quality controlled by BioAnalyzer. Six high-quality (RIN ≥ 9) RNA samples were randomly selected for NGS.

### miRNA and mRNA sequencing and pathway analysis

#### Illumina miRNA and mRNA Sequencing

Both miRNA and mRNA sequencing was performed on the Illumina HiSeq 2000 using RNA isolated from the same patient’s biopsy. NGS protocol is described in our previous study [14]. Briefly, sequencing was performed on the Illumina HiSeq 2000 single end 100 bp using the TruSeq SBS Kit v3-HS (Illumina, Inc, California, USA). For mRNA-seq, read mapping to human reference genome hg19 was done using Tophat 2.0.9. Counting the number of reads/gene was done using HTSeq v.0.5.4p3. The Bioconductor packages DESeq2 and edgeR (Bioconductor version: Release (3.2)) were used to identify differentially expressed genes. We set the threshold for FDR-adjusted *p* value to 0.15 in order to maximize the number of genes used to build the pathways. The miRNA Target Filter tool in IPA (IPA®, QIAGEN Redwood City, http://www.qiagen.com/ingenuity was used to associate miRNAs from our miRNA sequencing datasets with experimentally observed and predicted mRNA targets. The predicted relationships are from TargetScan (v7.0; targetscan.org) and the experimentally observed relationships are from TarBase.

#### Hierarchical clustering and heatmaps

Hierarchical clustering and the associated heatmaps for miRNA and mRNA sequencing data were generated with the function heatmap2 in the R package gplots or GENE-E R package. Pairwise correlation matrix between items was computed (based on Pearson correlation method) and then converted as a distance matrix and finally clustering was computed on the resulting distance matrix. Average linkage method used average to calculate the distance matrix. For the heatmap visualization, the log2-expression values were used.

#### Principal component analysis (PCA)

For a principal component analysis on log2 fold change of mRNA and microRNA expression of patients compared to controls, ‘prcomp’ function implemented in R (R Core Team, 2016), rgl and scatterplot3d R package was used for the principal component analysis of three-dimensions plots. To achieve a better numerical accuracy, the calculation was done by a singular value decomposition of the (centered and scaled) data matrix.

#### Gene and miRNA clouds (tag clouds, word clouds)

To condense and visualize gene enrichment data from a pathway analysis dataset a cloud was created using Wordle.net and Word cloud R package. The font size of a gene or miRNA (tag) is determined by its incidence in the pathway analysis dataset.

#### Functional enrichment analysis

Gene Ontology (GO) over-representation analysis (ORA) [15] methods were used to gain biological insight on the DEGs. We used clusterProfiler (version 3.18.1) package [16] in R to perform GO-ORA and GO-GSEA on biological process (BP) terms associated with DEGs. Results obtained at a threshold of *p* value below 0.1 were considered statistically significant.

#### QPCR validation of NGS studies

Total RNA was isolated from bladder dome biopsies using the miRVana miRNA isolation kit (Ambion). The reverse transcription reactions were carried out using the High Capacity cDNA Reverse Transcription Kit (Applied Biosystems) with random hexamer primers. TaqMan assays were from Applied Biosystems. QPCR was carried out in triplicates using 7900HT Fast Real-time PCR System (Applied Biosystems). The Ct values obtained after the real time-QPCR were normalized to 18S expression and fold differences compared to the average of controls calculated.

#### NanoString nCounter analysis

miRNA presence and content were analysed with the nCounter Human miRNA Expression Assay kit (NanoString, Seattle, WA) according to manufacturer's instructions with some modifications, as described in our earlier publication [17]. Briefly, 3 µl of each total RNA sample was used as input into the nCounter Human miRNA sample preparation. Hybridization was conducted for 12 h at 65 °C. Subsequently, the strip tubes were placed into the nCounter Prep Station for automated sample purification and subsequent reporter capture. Each sample was scanned for 600 FOV on the nCounter Digital Analyzer. The R Packages were used for NanoString Data Analysis: “NanoStringNorm” and “NanoStringDiff" (Available in CRAN). Background correction was performed based on the detected values of negative control probes, a within-sample normalization was calculated based on the observed values of positive control probes and normalization across samples using reference (housekeeping) genes. Additionally, we used a new algorithm called Removing Unwanted Variation-III (RUV-III) [18] which employs technical replicates and suitable control genes to normalise the data. “EdgeR" was used for expression profiling.

### Statistics

#### Hierarchical clustering and heatmaps

Hierarchical clustering and the associated heatmaps for miRNA and mRNA sequencing data were generated with the function heatmap2 in the R package gplots or GENE-E R package. Pairwise correlation matrix between items was computed based on Pearson correlation method. Average linkage method used average to calculate the distance matrix. For the heatmap visualization the log2-expression values were used. We used dendextend R package to create and compare visually appealing tree diagrams. *Principal Component analysis (PCA).* ‘Prcomp’ function implemented in R (R Core Team, 2016), rgl and scatterplot3d R package were used for the principal component analysis of three-dimensions plots. The calculation was done by a singular value decomposition of the (centered and scaled) data matrix. For *QPCR validation* the log2 fold change differences to the average of control samples were calculated. A one-way analysis of variance (ANOVA) was employed and the Tukey correction used to correct *p* values. The *p* value < 0.05 was considered statistically significant (GraphPad Prism (version 7.01)). *Contingency analysis.* Graphical matrix was drawn using the function balloonplot() in gplots package in R.

## Results

### Differentially expressed genes in BPS compared to control

**Fig. 1:**
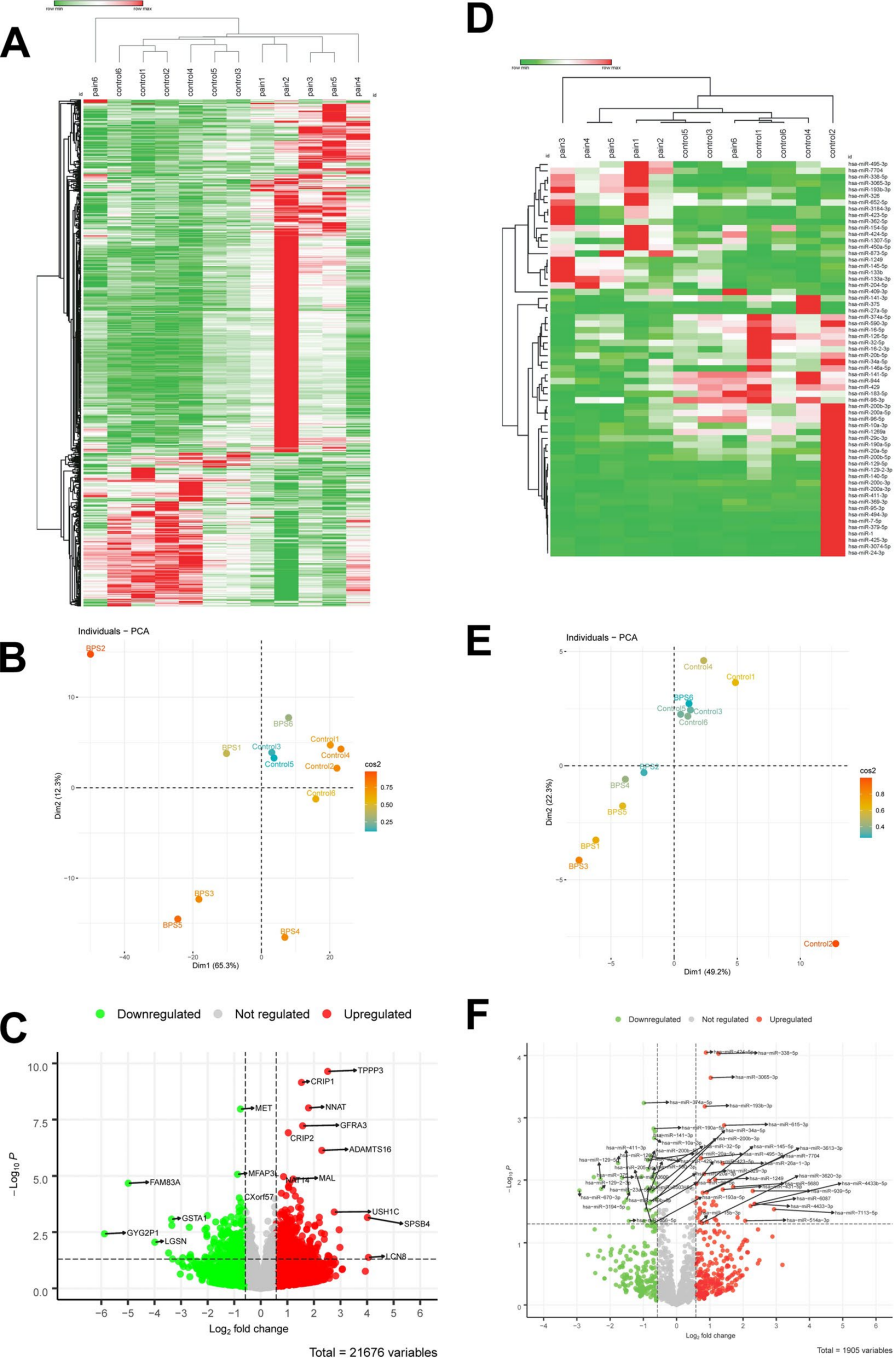
Differentially expressed mRNAs and miRNAs in BPS patients. **A** Heatmap and hieratical clustering based on normalized read counts of all mRNA (829) with *p* value < 0.05, absolute log2 fold change > 0.5 and mean of read counts > 50 reads. Genes are represented in y-axis and patients with BPS and controls are shown in x-axis. Normalized read counts were used for this heatmap. Minimum and maximum renormalized read counts for each mRNA were used to construct a relative color scheme by convert values to colors. One minus Pearson correlation metric was used for clustering accompanied with average linkage method. **B** Principal component analysis of the RNA sequencing data in a 2D graph of PC1 and PC2 based on normalized read counts of all mRNA (829) with *p* value < 0.05, absolute log2 fold change > 0.5 and mean of read counts > 50 reads. The bi-plot shows samples as labelled dots. Values of cos2 (square cosine, squared coordinates) for each sample indicate the quality of representation of the variables on the factor map. Samples are colour coded according to cos2 scale shown in the bar. **C** Volcano plot of all mRNAs. Up-regulated mRNAs are visualized in red color, and down-regulated in green. mRNAs with *p* value < 0.05, absolute log2 fold change > 0.5 were selected for further analysis. **D** Heatmap and hieratical clustering of all miRNA (62) with *p* value < 0.05, absolute log2 fold change > 0.5 and mean of read counts > 50 read. miRNAs are represented in y-axis and patients with BPS and controls are shown in x-axis. Normalized read counts were used for this heatmap. Minimum and maximum renormalized read counts for each miRNA were used to construct a relative color scheme by convert values to colors. One minus Pearson correlation metric was used for clustering accompanied with average linkage method. **E** Principal component analysis of the miRNA data in a 2D graph of PC1 and PC2. PC2 based on normalized read counts of all miRNA (62) with *p* value < 0.05, absolute log2 fold change > 0.5 and mean of read counts > 50 reads. The bi-plot shows samples as labelled dots. Samples are colour-coded according to the quality of representation of the variables on factor map (cos2, square cosine, squared coordinates) as shown on the side-bar. **F** Volcano plot of all miRNAs. All up-regulated miRNAs are visualized in red color, and all down-regulated in green. miRNAs with *p* value < 0.05, absolute log2 fold change > 0.5 were selected for further analysis

Differentially expressed genes (DEGs, mRNAs and miRNAs) were determined in BPS group using DESeq2 (with *p* value < 0.05, absolute log2 fold change > 0.5 and mean of read counts > 50 reads). Figure 1A shows the heatmap and hierarchical clustering based on normalized read counts of all significantly regulated mRNAs. Patients with BPS (“pain1-5”) cluster away from controls, although patient “pain6” more closely resembles controls in its DEGs profile. Selection of the 84 top regulated mRNAs (*p* value < 0.05, absolute log2 fold change > 1 and mean of read counts > 50 read) helped to cluster the two groups better (see the dendrogram in Additional file 8: Fig. S1A). PCA based on mRNAseq data shows good separation of BPS patients from controls (Fig. 1B). The scree plot in Additional file 8: Fig. S1B shows that component 1 and 2 can cover more than 75% of variation between samples. To characterize the relationship between 15 top regulated genes and each single patient in mRNA data set, a contingency analysis was performed. TPPP3, CRIP1, and MFAP5 showed a higher the probability of being indicative for BPS group (Additional file 8: Fig. S1C). DESeq2 analysis resulted in 829 differentially expressed mRNAs (Additional file 2). Overall, we identified 574 up-regulated and 255 down-regulated genes. Volcano plot of significantly regulated mRNAs based on *p* value (Fig. 1C) or adjusted *p* value (Additional file 8: Fig. S1) showed that TPPP3 and CRIP1 are the most up-regulated genes while, MET and FAM83A are most down-regulated genes.

The hierarchical clustering of samples based of differentially expressed miRNAs was very similar to the one based on mRNAs (Fig. 1D), likewise sample “pain6” was more similar to the controls than the rest of the samples. In totals, 62 miRNAs were changed during BPS, and PCA based on their normalized read counts clearly differentiated BPS from controls (Fig. 1E). The scree plot shown in Additional file 8: Fig. S2B shows that component 1 and 2 can cover more than 72% of variation between samples. The significant differentially expressed miRNAs are shown as a volcano plot (Fig. 1F). The background data to the miRNA analysis are shown in Additional file 8: Fig. S2.

### Functional enrichment analysis and identification of dysregulated biological processes in BPS

Using IPA, 31 significant pathways were built, though most did not have a defined z-score indicative of whether the activity of canonical pathways, including functional end-points, is increased or decreased based on DEGs in the datasets. Top 5 canonical IPA pathways based on mRNA datasets are shown in Table 1 (full list of pathways in Additional file 3). The two pathways involved in translational regulation, EIF2 Signalling and Regulation of eIF4 and p70S6K Signalling, are among the most significantly regulated processes during BPS. Fibrotic pathway important for bladder remodelling is also significant but without a z-score indicative of up- or down-regulation. Leukotriene Biosynthesis nociceptive pathway is significantly activated (positive z-score).

**Table 1:** Top 5 canonical IPA pathways regulated in BPS based on *p* value

Ingenuity canonical pathways	−log(*p* value)	Ratio	z-score	Molecules
EIF2 signalling	9.15	0.125	3.771	DDIT3 EIF2AK3 EIF3G FAU MAP2K2 RPL10A RPL13 RPL18 RPL18A RPL24 RPL27 RPL28 RPL29 RPL35 RPL36 RPL37 RPL38 RPLP1 RPLP2 RPS15 RPS15A RPS16 RPS19 RPS21 RPS4X RPS7 RPS9 RRAS
Hepatic fibrosis/hepatic stellate cell activation	4.54	0.0968	NA	CCL2 CCL21 CCN2 COL18A1 COL19A1 COL22A1 COL4A2 COL6A1 COL6A2 COL9A3 CXCL9 IGFBP4 MET MYL6 MYL9 STAT1 TIMP1 VEGFB
Leukotriene biosynthesis	3.24	0.308	2	DPEP2 GGT1 GGT5 LTC4S
Regulation of eIF4 and p70S6K Signalling	2.81	0.0828	NA	EIF3G FAU ITGA2 MAP2K2 RPS15 RPS15A RPS16 RPS19 RPS21 RPS4X RPS7 RPS9 RRAS
Cholesterol biosynthesis	2.69	0.172	− 2.236	HMGCR HMGCS1 MSMO1 SC5D SQLE

**Fig. 2:**
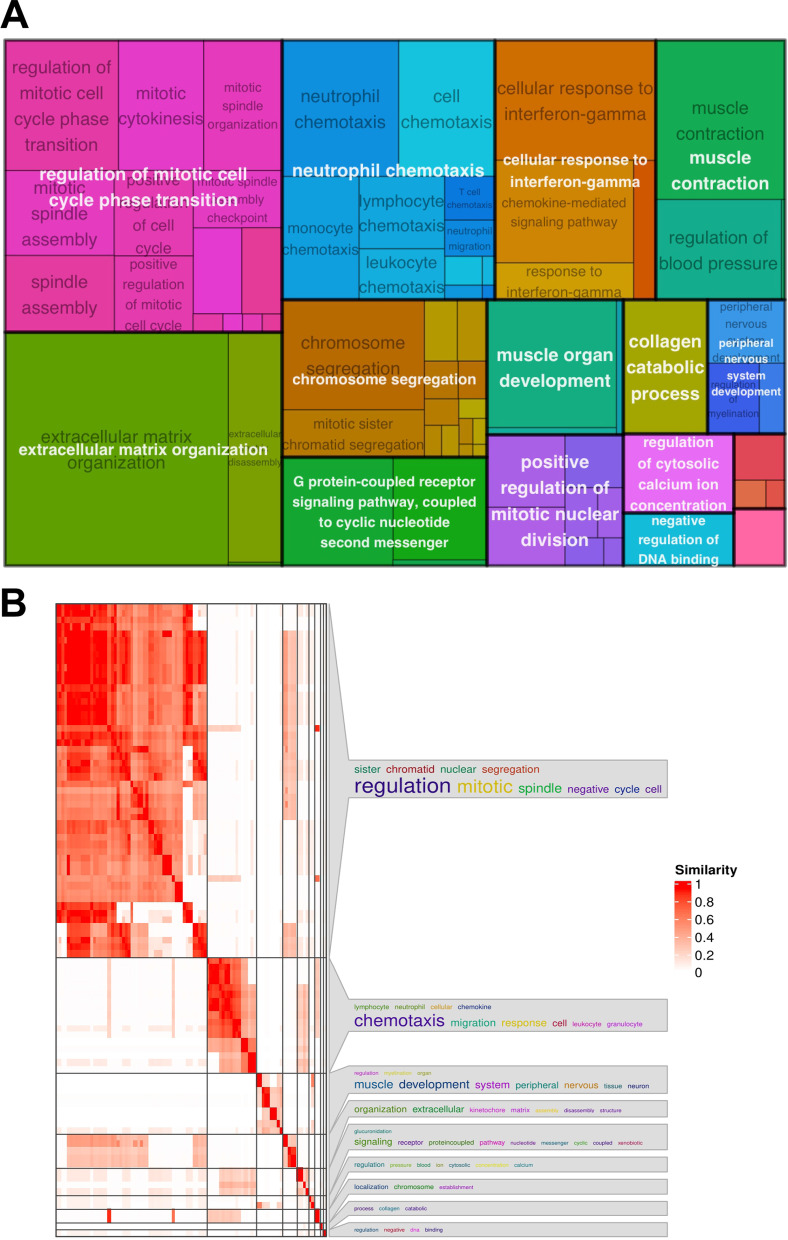
Gene ontology over-representation analysis of regulated mRNAs in BPS patients. **A** A treemap view of GO-term clusters, where each tile and colour represent a term and cluster, respectively. The list of GO terms was converted into a semantic similarity matrix using binary cut method. Tile size and group representatives of each cluster are corresponding to the GO terms’ size. **B** GO terms as clusters. Regulation of cell cycle, neutrophil chemotaxis and muscle and nervous system development are the top 3 clusters for BPS

**Fig. 3:**
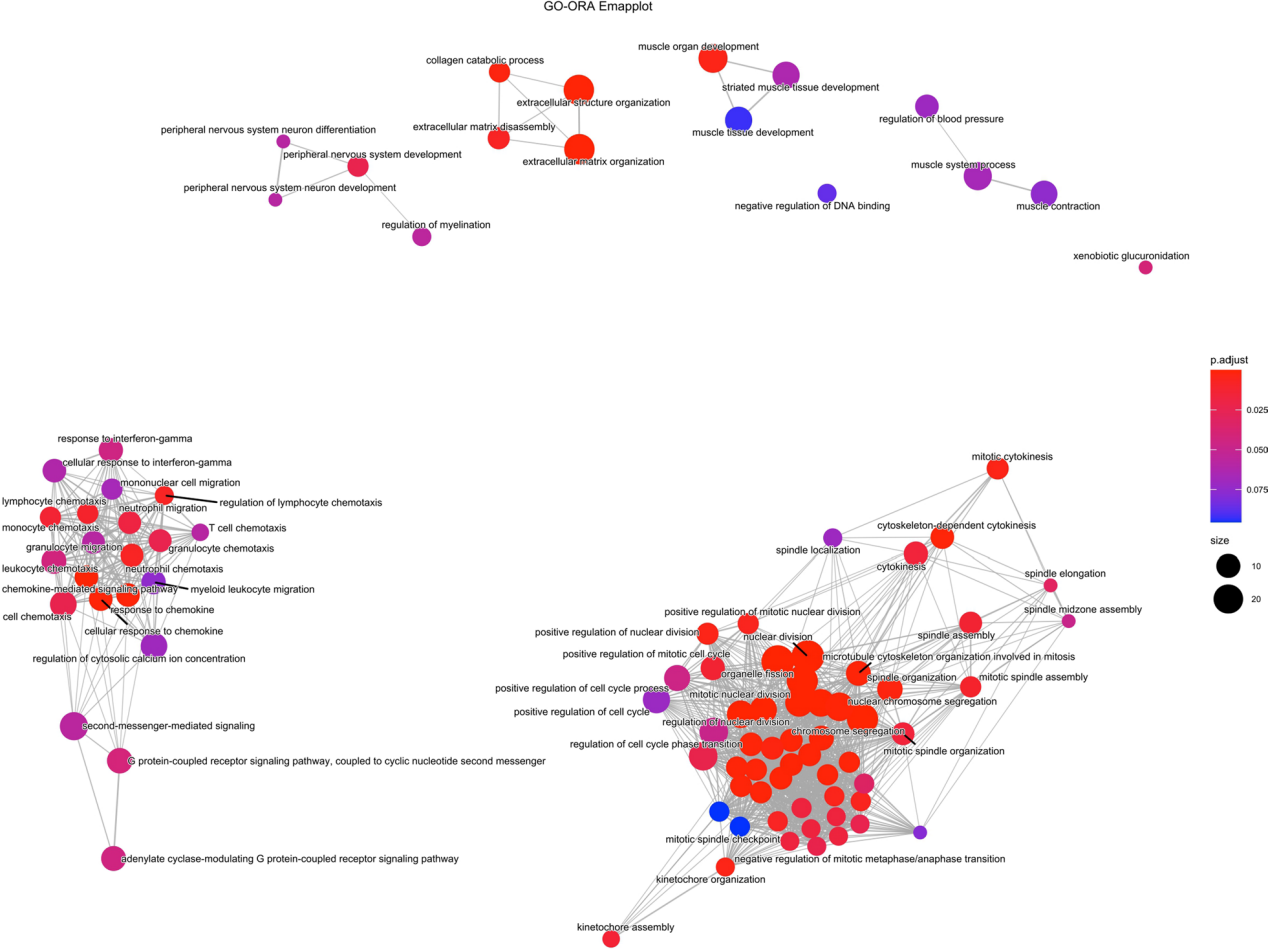
Enrichment Map plot of GO ORA (mRNA dataset). The network of enriched terms with edges connecting overlapping gene sets clusters mutually overlapping gene sets, visualizing the six main functional modules for BPS

**Fig. 4:**
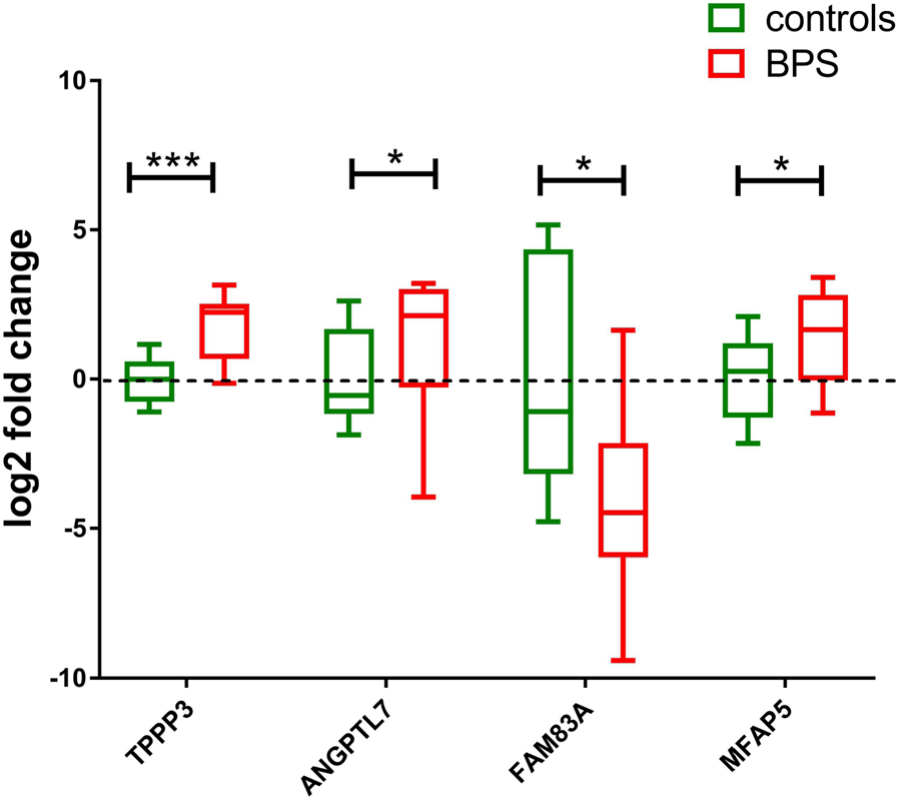
QPCR validation of selected DEGs based on NGS mRNA dataset. QPCR results for 4 mRNAs, regulated and significant (*p* < 0.05) in NGS mRNA dataset. Gene expression was determined in RNA samples isolated from controls (n = 8) and BPS patients’ biopsies (n = 16) using TaqMan gene expression The Ct values obtained after the real time-QPCR were normalized to 18S rRNA expression and fold differences compared to the average of controls calculated. Boxplot of log2 fold changes relative to the average normalized Ct values in the control group is shown. Statistically significant differences (*p < 0.05, ***p < 0.001, ANOVA)

In order to gain insight into the biological processes underlying BPS, we resorted to Gene Ontology (GO) Over Representation Analysis (ORA). The GO terms and associated genes, obtained from the ORA result are listed Additional file 4. To gain a better insight into the GO terms network, we created a semantic similarity matrix for a given list of GO terms depending on the information content of their closest common ancestor term. After removing the redundant GO terms based on semantic similarity score threshold of 0.7, remaining GO terms were grouped by clustering the semantic similarity matrix using binary cut method. A treemap view of GO-term clusters, where each tile and colour represent a term and cluster respectively, is shown in Fig. 2A. In the treemap plot, tile size and group representatives of each cluster corresponds to the GO terms’ size. Processes involved in cell division are highly represented in the BPS dataset, based on the abundance of mitosis-connected GO terms (Fig. 2A), followed by ECM reorganisation, neutrophil chemotaxis and muscle contractility. Notably, peripheral nervous system development is a prominent feature of this dataset. Figure 2B represents these GO terms as clusters, clustered by binary cut method. There, regulation of cell cycle, neutrophil chemotaxis and muscle and nervous system development are the top 3 clusters for BPS. We identified functional modules using Enrichment Map plot (Fig. 3). The network of enriched terms with edges connecting overlapping gene sets clustered mutually overlapping gene sets, visualizing the six main functional modules for BPS: cell cycle regulation, chemotaxis of immune cells, muscle development, muscle contraction, remodelling of extracellular matrix and peripheral nervous system organization and development (Fig. 3). To confirm the NGS results, we investigated the expression levels of 4 selected mRNAs, including the top regulated FAM83A and TPPP3 (Fig. 1C) in the bladder biopsies of a larger independent patient cohort (n = 8 controls and n = 16 BPS). mRNAs for TPPP3, ANGPTL17 and MFAP5 were significantly up-regulated (*p* < 0.05), whereas FAM83A was down-regulated in BPS, in accordance with the NGS data (Fig. 4).

### Identification of functionally important miRNAs altered in BPS dataset

**Fig. 5:**
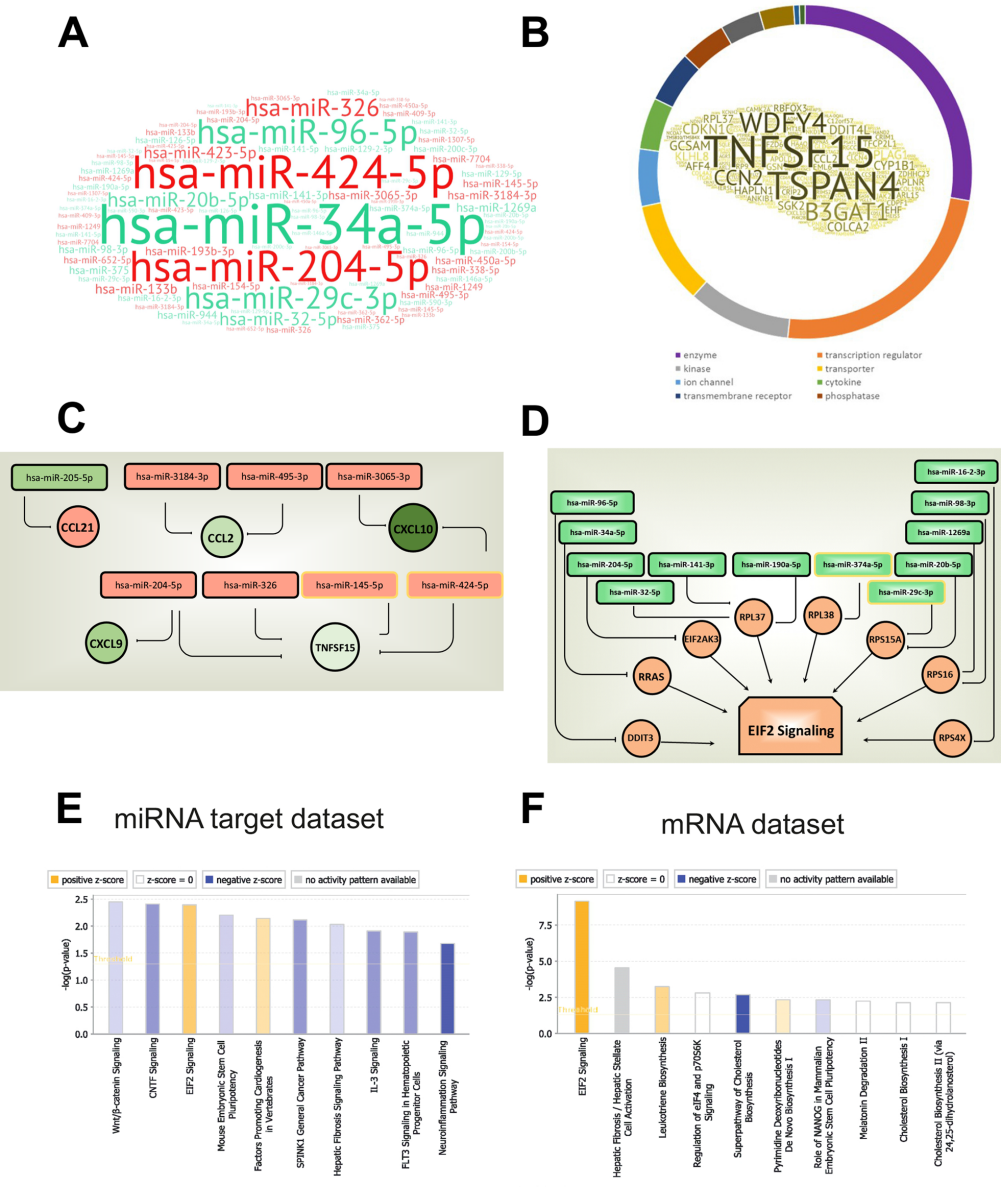
Pathway analysis using mRNAs, targeted by miRNAs regulated in BPS. **A** Role of miRNAs depending on the number of their appropriately regulated targets. Word cloud of up-regulated (in red) and down-regulated (in green) miRNAs, font size corresponding to the number of correctly regulated mRNAs, targeted by each specific miRNA. **B** Word cloud of mRNAs, targeted by dysregulated miRNAs; font size corresponds to the number of miRNAs affecting the expression of a particular mRNA. The coloured circle shows the functional groups of the affected mRNAs: most are enzymes and transcription regulators, kinases and cytokines. **C** Cytokines up-regulated (red) and down-regulated (green) and the targeting miRNAs. Note that some cytokines are affected by several miRNAs, and some miRNAs regulate multiple cytokine mRNAs. **D** EIF2 signalling pathway might be activated due to the down-regulation of miRNAs, targeting its elements. Down-regulated miRNAs are shown in green, up-regulated target genes in red. **E** Pathway analysis based on genes, predicted to be targeted by dysregulated miRNAs and differentially expressed in BPS. Top 10 IPA Pathways are shown (based on *p* value). **F** Pathway analysis based on all regulated mRNAs in BPS, top 10 pathways are shown

We identified 62 microRNAs, regulated and abundant in BPS (Additional file 5) and determined which of their target mRNAs were present and correctly regulated in the mRNA dataset (Additional file 6). The word cloud in Fig. 5A illustrates the miRNAs, with the font size reflecting the number of correctly regulated target mRNAs. Up-regulated miR-424-5p and down-regulated miR-34a-5p have 40 and 45 regulated target genes in mRNA dataset, respectively, and potentially play the highest regulatory role in BPS. Word cloud of the miRNA targets includes TNFSF15, TSPAN4 and WDFY4 mRNAs which are targeted by multiple miRNAs (Fig. 5B). MiRNA-regulated genes fall into multiple categories, the majority being enzymes, transcriptional regulators, transporters and kinases.

Multiple cytokines differentially expressed in the BPS dataset are paired with their regulatory miRNAs (Fig. 5C): CCL21 is increased, while miR-205-5p which is predicted to target it, is down-regulated; the same correct behaviour was observed for the down-regulated CCL2, CXCL10, CXCL9, whose regulatory miRNAs were all increased (Fig. 5C). Similarly, the up-regulated components of the activated EIF2 signalling pathway are regulated by miRNAs which are decreased in the biopsies of patients with BPS (Fig. 5D). Top ten significant IPA pathways built using appropriately regulated targets of miRNAs are shown in Fig. 5E. Like in the mRNA dataset shown in Fig. 5F for comparison, eIF2 signalling is the main activated pathway, targeted by miRNAs regulated in BPS.

To validate the miRNA expression results, we carried out a profiling study using NanoString nCounter Human miRNA Expression Assay kit (Additional file 7) using n = 7 controls and n = 16 BPS samples. Comparative regulation of 16 miRNAs, robustly detected by NanoString in all samples, is shown in Additional file 8: Fig. S3. We confirmed the down-regulation of miR-205-5p, and up-regulation of muscle-specific miR-145-5p, miR-143-3p and miR-133a-3p in BPS patients.

## Discussion

IC/BPS is a chronic disease of unknown etiology, it is a multifactorial, heterogeneous disorder with several clinical phenotypes. A variety of pathophysiological origins have been proposed for BPS, ranging from autoimmunity, inflammation caused by recurrent bladder injury, neurogenic inflammation with the contributing increase of urothelial permeability and disruption of the GAG layer, and altered central nociception [4, 5]. Emerging gene expression studies, using human tissue (bladder biopsies or urine sediment) or tissue from animal models of IC have shed some light on bladder alterations during BPS, however, no consensus exists regarding the molecular drivers of this dysfunction. Recent findings indicate that IC/BPS with Hunner’s lesions is a distinct histological and molecular phenotype of the disease, characterized by pancystitis, frequent clonal B-cell expansion and epithelial denudation [19]. Therefore, current guidelines [20] suggest that Hunner-type interstitial cystitis and bladder pain syndrome should be considered separate entities, because bladder pain syndrome shows minimal pathological changes in the bladder [20]. While the gene expression profile of Hunner-type IC seems quite distinct from the controls, both in the biopsies and urine sediment [11, 12], the patients with non-ulcerative BPS/IC are less clearly definable, and often show no or slight differences in gene expression compared to control either in the biopsies [12], or urine sediment [9].

In this study we analyzed the NGS mRNA and miRNA gene expression profiles in the bladder dome biopsies of female patients with BPS. Unlike many earlier gene expression studies, performed using low throughput methodology (microarrays, PCR arrays with selected genes), we carried out a comprehensive paired mRNA and miRNA transcriptome analysis from the same patient, allowing us to discern the involvement of particular miRNA targets in the signalling events. The patient group selected in our trial was not specifically recruited based on the presence of Hunner’s ulcers, our patients did not have the ulcerative phenotype, and  therefore we refer to them as BPS. Most patients in our study had a low bladder capacity with median cystometric bladder volume of 200 ml, and a measurable infiltration of neutrophils and mast cells in the bladder submucosa. The NGS mRNA and miRNA gene expression profiles in our BPS patients (n = 6) were distinct from the controls. This is in agreement with previous findings, pointing to the correlation of gene expression with bladder capacity [8], where the patients with normal bladder capacity were similar to controls, and patients whose bladder volume ranged from 300 to 175 ml were significantly different.

We could use the DEGs mRNA dataset to build IPA pathways, though only a limited number of significant pathways could be assigned a z-score. Nevertheless, two out of top 5 pathways were involved in translational regulation. EIF2 Signalling and Regulation of eIF4 and p70S6K Signalling are among the most significantly regulated processes during BPS. They are activated in response to cellular stress and regulate proinflammatory cytokine expression [21]. Importantly, both pathways were previously described in the urine sediment cells from BPS patients [11], highlighting the importance of cellular stress in the pathogenesis of BPS. Fibrotic pathway appears to be involved, pointing to organ remodelling. Leukotriene Biosynthesis nociceptive pathway, important in a variety of allergic/inflammatory diseases and in neuropathic pain [22], is also significantly activated (positive z-score). Leukotrienes are produced and released by neutrophils and mast cells, which are increased in our population of BPS patients. Mast cell marker tryptase (TPSAB1 gene) was strongly and significantly upregulated in the BPS NGS dataset (Log2 fold change (log2 FC) of 1.16, *p* value 0.004).

Previously, attempts have been made to distinguish BPS/IC from OAB using immunohistochemistry with antibodies to the nerve cell marker PGP9.5 (neuron-specific protein gene product 9.5), p75NTR (p75 neurotrophin receptor), the B-lymphocyte marker CD20 and mast cell tryptase [23]. Clinically, patients with OAB are similar to patients with BPS/IC regarding frequency and urgency as well as low bladder capacity but usually do not suffer from pain. In addition to the increase of tryptase mRNA, we observed an up-regulation of PGP9.5 gene UCHL1 (log2 FC 0.56), and p75 neurotrophin receptor gene NTRK1 (log2 FC 1.24) in our dataset, indicative of sensory hyperinnervation in BPS. Similarly, several inflammatory mediators, identified earlier by QPCR in trigone biopsies of BPS patients using TaqMan microfluidic cards [10], showed similar regulation of expression: CCL21 (log2 FC 1.15, *p* value 0.01) and CXCL1 (log2 FC 0.76) mRNAs were both significantly up-regulated in our NGS dataset. We observed that many regulatory miRNAs, altered during BPS, were involved in the regulation of inflammatory mediators, and that the levels of CCL21 and its regulatory miR-205-5p were inversely correlated. Chemokine CCL21 activates microglia in the central nervous system and is expressed in neurons after an insult or mechanical injury, implicated in neuropathic pain [24], rheumatoid arthritis [25] and pulmonary fibrosis [26]. CCR7, the receptor for CCL21, was also up-regulated in the NGS dataset (log2 FC 0.66, n.s.), so it is possible that at least in some BPS patients, the relevant signalling cascade is activated, contributing to the activation of the ERK and JNK pathways. Recent studies have linked bladder inflammation with mood disorders [27]. Although we did not specifically examine this, it is tempting to speculate that the up-regulation of CCL21 reported here might have a bearing on the higher than average incidence of depression, common in BPS [28, 29].

We performed GO ORA using DEGs from the BPS mRNAseq dataset to reveal the biological processes contributing to bladder remodelling. The pathways were clustered into six main functional groups: cell cycle regulation, chemotaxis of immune cells, muscle development, muscle contraction, remodelling of extracellular matrix and peripheral nervous system organization and development. Generally, immune pathways activation, which was a prominent feature of BOO-induced bladder overactivity, and BPH-induced bladder remodelling [14] was modest in our dataset, limiting itself to neutrophil chemotaxis and IFN-γ-mediated signalling. Our data support the notion that, unlike Hunner-type IC, inflammation is less pronounced in non-ulcerative BPS, although it might play an important role in disease pathogenesis. Activation of mitosis, catabolic connective tissue remodelling and muscle tissue development, observed on the molecular level in our dataset, are in line with the bladder morphology of the majority of our subjects, who had thick-walled, low capacity bladders.

Our study has its limitations: only female patients have been recruited; controls for QPCR were also female, but NGS controls included 4 male subjects. To overcome the last limitation, the expression of the Y chromosome-linked genes was not considered in the DEG analysis. The study has a relatively small number of patients, which might have a bearing on our results, given the heterogeneity of BPS.

In conclusion, we studied the gene expression profiles of mRNA and miRNA in bladder biopsies of BPS patients, and identified the key biological functions, contributing to organ remodelling. Non-ulcerative BPS patients, recruited in this study, had strong smooth muscle contractile and cell proliferative phenotype, indicative of smooth muscle hyperplasia. The contribution of inflammatory processes was modest, but we observed molecular changes indicative of neuroinflammation and peripheral nervous system re-organization and development. Pathway analysis of the integrated mRNA-miRNA NGS dataset shed some light on the molecular changes in the bladders of patients with BPS, and pinpointed important regulatory miRNAs whose dysregulation might contribute to the pathogenesis.

## Supplementary Information

**Additional file 1**. Demographical and clinical information about the subjects participating in the study.

**Additional file 2.** List of differentially expressed genes with p-value<0.05, absolute log2 fold change>0.5 and mean of read counts>50 reads.

**Additional file 3.** List of significant Ingenuity Canonical Pathways

**Additional file 4.** Gene Ontology (GO) terms and associated genes, obtained from the Over Representation Analysis (ORA) with adjusted p value < 0.1

**Additional file 5.** List of abundant microRNAs, significantly regulated in the BPS patients compared to controls

**Additional file 6.** List of the mRNA targets of 62 miRNAs, abundant and regulated in BPS

**Additional file 7.** All data on miRNAs, detected in the profiling using NanoString nCounter Human miRNA Expression Assay kit

**Additional file 8: Supplementary Figures. Fig. S1**. Characterization of regulated mRNAs. (A) Sample clustering based on the expression of the 84 top regulated mRNAs (p value<0.05, absolute log2 fold change>1 and mean of read counts>50 read). (B) Scree plot for visualization of the percentage of variances explained for each principle component (eigenvalues) in RNA sequencing data (C) Contingency table of the 18 top regulated mRNAs displaying the (multivariate) frequency distribution of the variables. The diameter of the orange circle for each gene per patient provides an estimate of the probability for that particular gene to represent that particular patient. (D) Volcano plot of all mRNAs using adjusted P value. **Fig. S2**. Characterization of regulated miRNAs. (A) Sample clustering based on the expression of the 18 top regulated miRNAs (p-value<0.05, absolute log2 fold change>1 and mean of read counts>50 read) (B) Scree plot for visualization of the variances in miRNA sequencing data (C) Contingency table of the 8 top regulated miRNAs displaying the (multivariate) frequency distribution of the variables. The diameter of the orange circle for each miRNA per patient provides an estimate of the probability for that particular miRNA to represent that particular patient. (D) Volcano plot of all miRNAs using adjusted P value. **Fig. S3**. Validation of regulated miRNAs by NanoString. Radar graph visualizing the average log2 fold change of 16 miRNAs significantly regulated in NanoString or sequencing dataset (p-value<0.05, absolute log2 fold change>1 and mean of read counts>300 read). The red line represents the NGS miRNA sequencing data and purple line represents the NanoString data. Red area is showing the upregulation and green area is representing downregulation.

## Data Availability

The mRNA- and miRNA-seq datasets were deposited in the European Nucleotide Archive (ENA) under ENA accession numbers: PRJEB46961 for mRNA and PRJEB10955 for miRNA. The lists of genes used for downstream analysis and regulated genes involved in signalling pathways are available as Additional files.

## Abbreviations

IC/BPS: Interstitial cystitis/bladder pain syndrome
NGS: Next-generation sequencing
DEGs: Differentially expressed genes
mRNA: Messenger RNA
miRNA: MicroRNA
GO ORA: Gene ontology over-representation analysis
QPCR: Quantitative real-time polymerase chain reaction
eIF2(4): Eukaryotic Initiation Factor 2 (4)
FC: Fold change
n.s.: Not significant

## References

[1] van de Merwe JP, Nordling J, Bouchelouche P, Bouchelouche K, Cervigni M, Daha LK (2008). Diagnostic criteria, classification, and nomenclature for painful bladder syndrome/interstitial cystitis: an ESSIC proposal. EurUrol.

[2] Clemens JQ, Meenan RT, Rosetti MC, Gao SY, Calhoun EA. Prevalence and incidence of interstitial cystitis in a managed care population. J Urol. 2005;173(1):98–102; discussion 10.1097/01.ju.0000146114.53828.82. 10.1097/01.ju.0000146114.53828.82 15592041

[3] Berry SH, Elliott MN, Suttorp M, Bogart LM, Stoto MA, Eggers P (2011). Prevalence of symptoms of bladder pain syndrome/interstitial cystitis among adult females in the United States. J Urol.

[4] Karamali M, Shafabakhsh R, Ghanbari Z, Eftekhar T, Asemi Z (2019). Molecular pathogenesis of interstitial cystitis/bladder pain syndrome based on gene expression. J Cell Physiol.

[5] Akiyama Y, Luo Y, Hanno PM, Maeda D, Homma Y (2020). Interstitial cystitis/bladder pain syndrome: the evolving landscape, animal models and future perspectives. Int J Urol.

[6] Sanchez-Freire V, Blanchard MG, Burkhard FC, Kessler TM, Kellenberger S, Monastyrskaya K (2011). Acid-sensing channels in human bladder: expression, function and alterations during bladder pain syndrome. J Urol.

[7] Monastyrskaya K, Babiychuk EB, Draeger A, Burkhard FC (2013). Down-regulation of annexin A1 in the urothelium decreases cell survival after bacterial toxin exposure. J Urol.

[8] Colaco M, Koslov DS, Keys T, Evans RJ, Badlani GH, Andersson KE (2014). Correlation of gene expression with bladder capacity in interstitial cystitis/bladder pain syndrome. J Urol.

[9] Blalock EM, Korrect GS, Stromberg AJ, Erickson DR (2012). Gene expression analysis of urine sediment: evaluation for potential noninvasive markers of interstitial cystitis/bladder pain syndrome. J Urol.

[10] Offiah I, Didangelos A, Dawes J, Cartwright R, Khullar V, Bradbury EJ (2016). The expression of inflammatory mediators in bladder pain syndrome. Eur Urol.

[11] Izquierdo L, Mateu L, Lozano JJ, Montalbo R, Ingelmo-Torres M, Gómez A (2020). Urine gene expression profiles in bladder pain syndrome patients treated with triamcinolone. Eur Urol Focus.

[12] Akiyama Y, Maeda D, Katoh H, Morikawa T, Niimi A, Nomiya A (2019). Molecular taxonomy of interstitial cystitis/bladder pain syndrome based on whole transcriptome profiling by next-generation RNA sequencing of bladder mucosal biopsies. J Urol.

[13] Sanchez Freire V, Burkhard FC, Kessler TM, Kuhn A, Draeger A, Monastyrskaya K (2010). MicroRNAs may mediate the down-regulation of neurokinin-1 receptor in chronic bladder pain syndrome. Am J Pathol.

[14] Gheinani AH, Kiss B, Moltzahn F, Keller I, Bruggmann R, Rehrauer H (2017). Characterization of miRNA-regulated networks, hubs of signaling, and biomarkers in obstruction-induced bladder dysfunction. JCI insight.

[15] Boyle EI, Weng S, Gollub J, Jin H, Botstein D, Cherry JM (2004). GO::TermFinder–open source software for accessing Gene Ontology information and finding significantly enriched Gene Ontology terms associated with a list of genes. Bioinformatics.

[16] Yu G, Wang LG, Han Y, He QY (2012). clusterProfiler: an R package for comparing biological themes among gene clusters. OMICS.

[17] Gheinani AH, Vogeli M, Baumgartner U, Vassella E, Draeger A, Burkhard FC (2018). Improved isolation strategies to increase the yield and purity of human urinary exosomes for biomarker discovery. Sci Rep.

[18] Molania R, Gagnon-Bartsch JA, Dobrovic A, Speed TP (2019). A new normalization for Nanostring nCounter gene expression data. Nucleic Acids Res.

[19] Maeda D, Akiyama Y, Morikawa T, Kunita A, Ota Y, Katoh H (2015). Hunner-type (classic) interstitial cystitis: a distinct inflammatory disorder characterized by pancystitis, with frequent expansion of clonal B-cells and epithelial denudation. PLoS ONE.

[20] Homma Y, Akiyama Y, Tomoe H, Furuta A, Ueda T, Maeda D (2020). Clinical guidelines for interstitial cystitis/bladder pain syndrome. Int J Urol.

[21] Shrestha N, Bahnan W, Wiley DJ, Barber G, Fields KA, Schesser K (2012). Eukaryotic initiation factor 2 (eIF2) signaling regulates proinflammatory cytokine expression and bacterial invasion. J Biol Chem.

[22] Noguchi K, Okubo M (2011). Leukotrienes in nociceptive pathway and neuropathic/inflammatory pain. Biol Pharm Bull.

[23] Regauer S, Gamper M, Fehr MK, Viereck V (2017). Sensory hyperinnervation distinguishes bladder pain syndrome/interstitial cystitis from overactive bladder syndrome. J Urol.

[24] Honjoh K, Nakajima H, Hirai T, Watanabe S, Matsumine A (2019). Relationship of inflammatory cytokines from M1-Type microglia/macrophages at the injured site and lumbar enlargement with neuropathic pain after spinal cord injury in the CCL21 knockout (plt) mouse. Front Cell Neurosci.

[25] Pickens SR, Chamberlain ND, Volin MV, Pope RM, Talarico NE, Mandelin AM (2012). Role of the CCL21 and CCR7 pathways in rheumatoid arthritis angiogenesis. Arthritis Rheum.

[26] Pierce EM, Carpenter K, Jakubzick C, Kunkel SL, Flaherty KR, Martinez FJ (2007). Therapeutic targeting of CC ligand 21 or CC chemokine receptor 7 abrogates pulmonary fibrosis induced by the adoptive transfer of human pulmonary fibroblasts to immunodeficient mice. Am J Pathol.

[27] Hirshman NA, Hughes FM, Jin H, Harrison WT, White SW, Doan I (2020). Cyclophosphamide-induced cystitis results in NLRP3-mediated inflammation in the hippocampus and symptoms of depression in rats. Am J Physiol Renal Physiol.

[28] Crawford A, Tripp DA, Nickel JC, Carr L, Moldwin R, Katz L et al. Depression and helplessness impact interstitial cystitis/bladder pain syndrome pain over time. Canadian Urological Association journal = Journal de l'Association des urologues du Canada. 2019;13(10):328–33. 10.5489/cuaj.5703. 10.5489/cuaj.5703 PMC6788912 31364973

[29] McKernan LC, Walsh CG, Reynolds WS, Crofford LJ, Dmochowski RR, Williams DA (2018). Psychosocial co-morbidities in Interstitial Cystitis/Bladder Pain syndrome (IC/BPS): a systematic review. Neurourol Urodyn.

