# Supplementary material for: Integrated mRNA-miRNA transcriptome analysis of bladder biopsies from patients with bladder pain syndrome identifies signaling alterations contributing to the disease pathogenesis
Source: BMC Urol. 2021 Dec 7;21:172. doi: 10.1186/s12894-021-00934-0 (PMC8653529; doi:10.1186/s12894-021-00934-0)
Supplement: Supplementary file 8 — Additional file 8: Supplementary Figures. Fig. S1. Characterization of regulated mRNAs. (A) Sample clustering based on the expression of the 84 top regulated mRNAs (p value<0.05, absolute log2 fold change>1 and mean of read counts>50 read). (B) Scree plot for visualization of the percentage of variances explained for each principle component (eigenvalues) in RNA sequencing data (C) Contingency table of the 18 top regulated mRNAs displaying the (multivariate) frequency distribution of the variables. The diameter of the orange circle for each gene per patient provides an estimate of the probability for that particular gene to represent that particular patient. (D) Volcano plot of all mRNAs using adjusted P value. Fig. S2. Characterization of regulated miRNAs. (A) Sample clustering based on the expression of the 18 top regulated miRNAs (p-value<0.05, absolute log2 fold change>1 and mean of read counts>50 read) (B) Scree plot for visualization of the variances in miRNA sequencing data (C) Contingency table of the 8 top regulated miRNAs displaying the (multivariate) frequency distribution of the variables. The diameter of the orange circle for each miRNA per patient provides an estimate of the probability for that particular miRNA to represent that particular patient. (D) Volcano plot of all miRNAs using adjusted P value. Fig. S3. Validation of regulated miRNAs by NanoString. Radar graph visualizing the average log2 fold change of 16 miRNAs significantly regulated in NanoString or sequencing dataset (p-value<0.05, absolute log2 fold change>1 and mean of read counts>300 read). The red line represents the NGS miRNA sequencing data and purple line represents the NanoString data. Red area is showing the upregulation and green area is representing downregulation. [file 12894_2021_934_MOESM8_ESM.pdf]

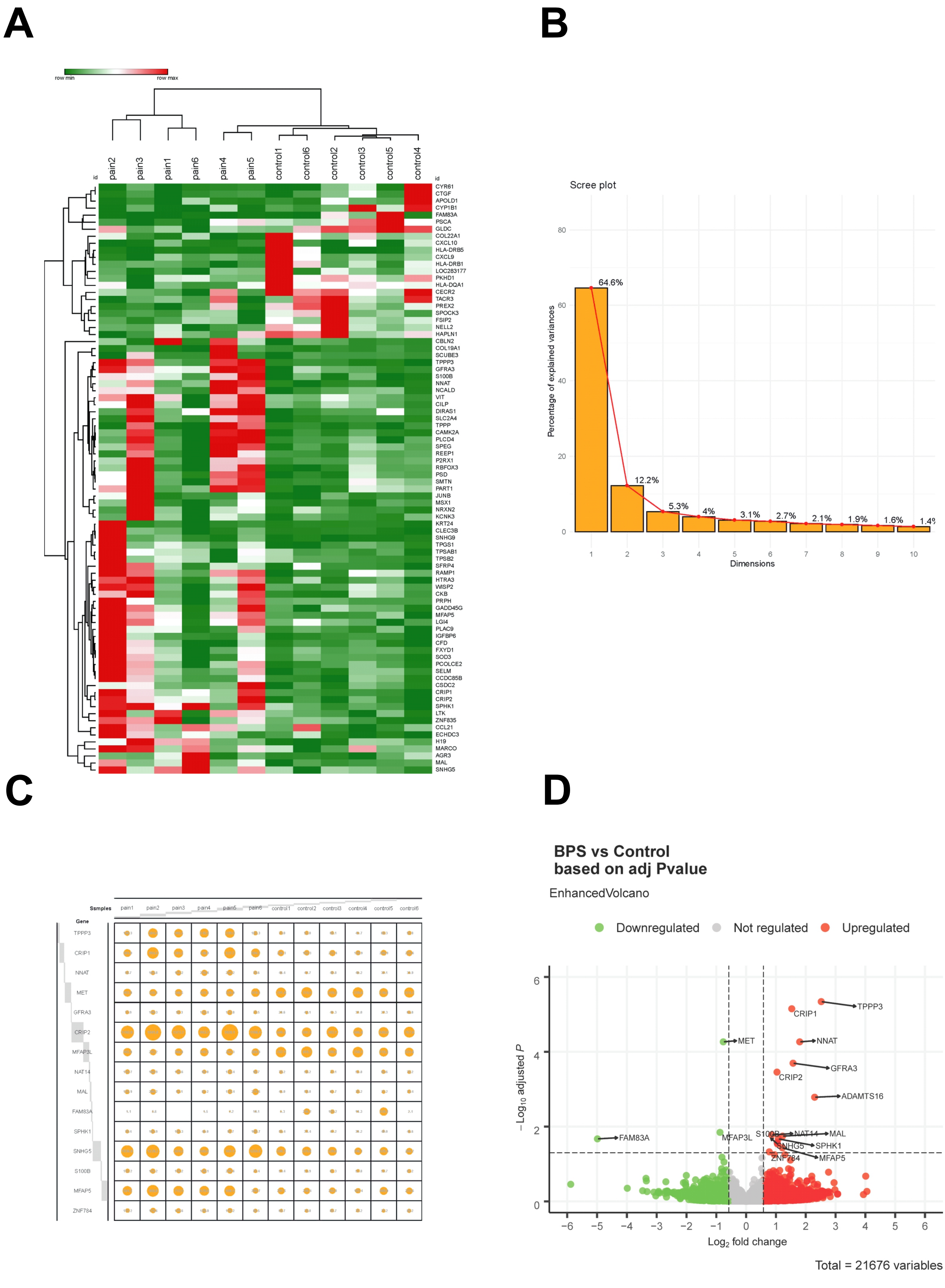
A
row min
row max
id
pain2
pain3
pain1
pain6
pain4
pain5
control1
control6
control2
control3
control5
control4
CYR61
CTGF
APOLD1
CYP1B1
FAM83A
PSCA
GLDC
COL22A1
CXCL10
HLA-DRB5
CXCL9
HLA-DRB1
LOC283177
PKHD1
HLA-DQA1
CECR2
TACR3
PREX2
SPOCK3
FSIP2
NELL2
HAPLN1
CBLN2
COL19A1
SCUBE3
TPPP3
GFRA3
S100B
NNAT
NCALD
VIT
CILP
DIRAS1
SLC2A4
TPPP
CAMK2A
PLCD4
SPEG
REEP1
P2RX1
RBFOX3
PSD
SMTN
PART1
JUNB
MSX1
NRXN2
KCNK3
KRT24
CLEC3B
SNHG9
TPGS1
TPSAB1
TPSB2
SFRP4
RAMP1
HTRA3
WISP2
CKB
PRPH
GADD45G
MFAP5
LGI4
PLAC9
IGFBP6
CFD
FXYD1
SOD3
PCOLCE2
SELM
CCDC85B
CSDC2
CRIP1
CRIP2
SPHK1
LTK
ZNF835
CCL21
ECHDC3
H19
MARCO
AGR3
MAL
SNHG5
B
Scree plot
Percentage of explained variances
Dimensions
64.6%
12.2%
5.3%
4%
3.1%
2.7%
2.1%
1.9%
1.6%
1.4%
C
Samples
Gene
pain1
pain2
pain3
pain4
pain5
pain6
control1
control2
control3
control4
control5
control6
TPPP3
CRIP1
NNAT
MET
GFRA3
CRIP2
MFAP3L
NAT14
MAL
FAM83A
SPHK1
SNHG5
S100B
MFAP5
ZNF784
D
BPS vs Control
based on adj Pvalue
EnhancedVolcano
Downregulated
Not regulated
Upregulated
$-Log_{10}$ adjusted P
Log_2 fold change
TPPP3
CRIP1
MET
NNAT
GFRA3
CRIP2
ADAMTS16
FAM83A
MFAP3L
S100B
NAT14
MAL
SNHG5
SPHK1
ZNF784
MFAP5
Total = 21676 variables

Fig. S1

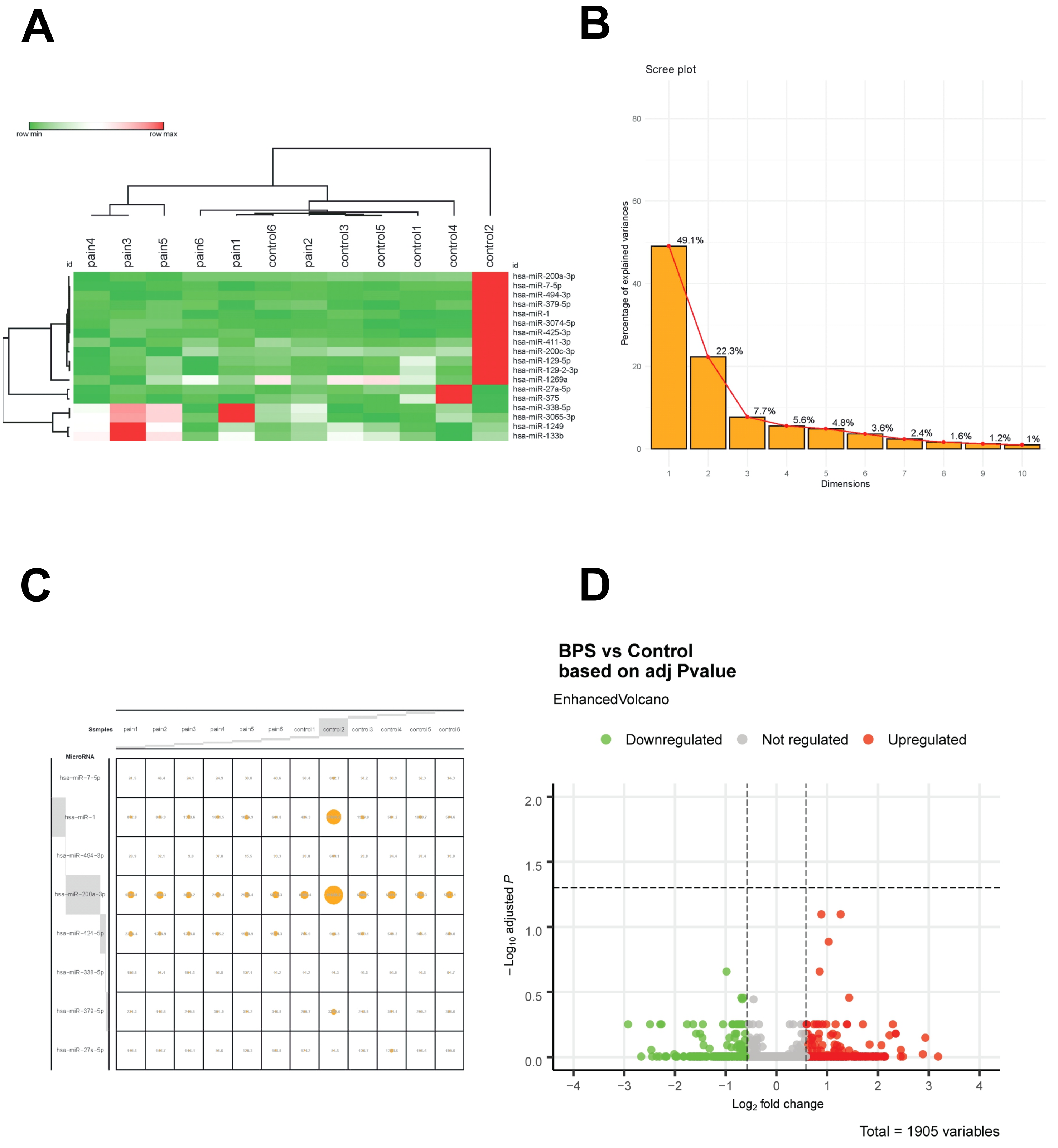
A
row min
row max
id
pain4
pain3
pain5
pain6
pain1
control6
pain2
control3
control5
control1
control4
control2
hsa-miR-200a-3p
hsa-miR-7-5p
hsa-miR-494-3p
hsa-miR-379-5p
hsa-miR-1
hsa-miR-3074-5p
hsa-miR-425-3p
hsa-miR-411-3p
hsa-miR-200c-3p
hsa-miR-129-5p
hsa-miR-129-2-3p
hsa-miR-1269a
hsa-miR-27a-5p
hsa-miR-375
hsa-miR-338-5p
hsa-miR-3065-3p
hsa-miR-1249
hsa-miR-133b
B
Scree plot
Percentage of explained variances
Dimensions
49.1%
22.3%
7.7%
5.6%
4.8%
3.6%
2.4%
1.6%
1.2%
1%
C
Ssmples
pain1
pain2
pain3
pain4
pain5
pain6
control1
control2
control3
control4
control5
control6
MicroRNA
hsa-miR-7-5p
hsa-miR-1
hsa-miR-494-3p
hsa-miR-200a-3p
hsa-miR-424-5p
hsa-miR-338-5p
hsa-miR-379-5p
hsa-miR-27a-5p
D
BPS vs Control
based on adj Pvalue
EnhancedVolcano
Downregulated
Not regulated
Upregulated
$-Log_{10}$ adjusted P
Log_2 fold change
Total = 1905 variables

Fig. S2

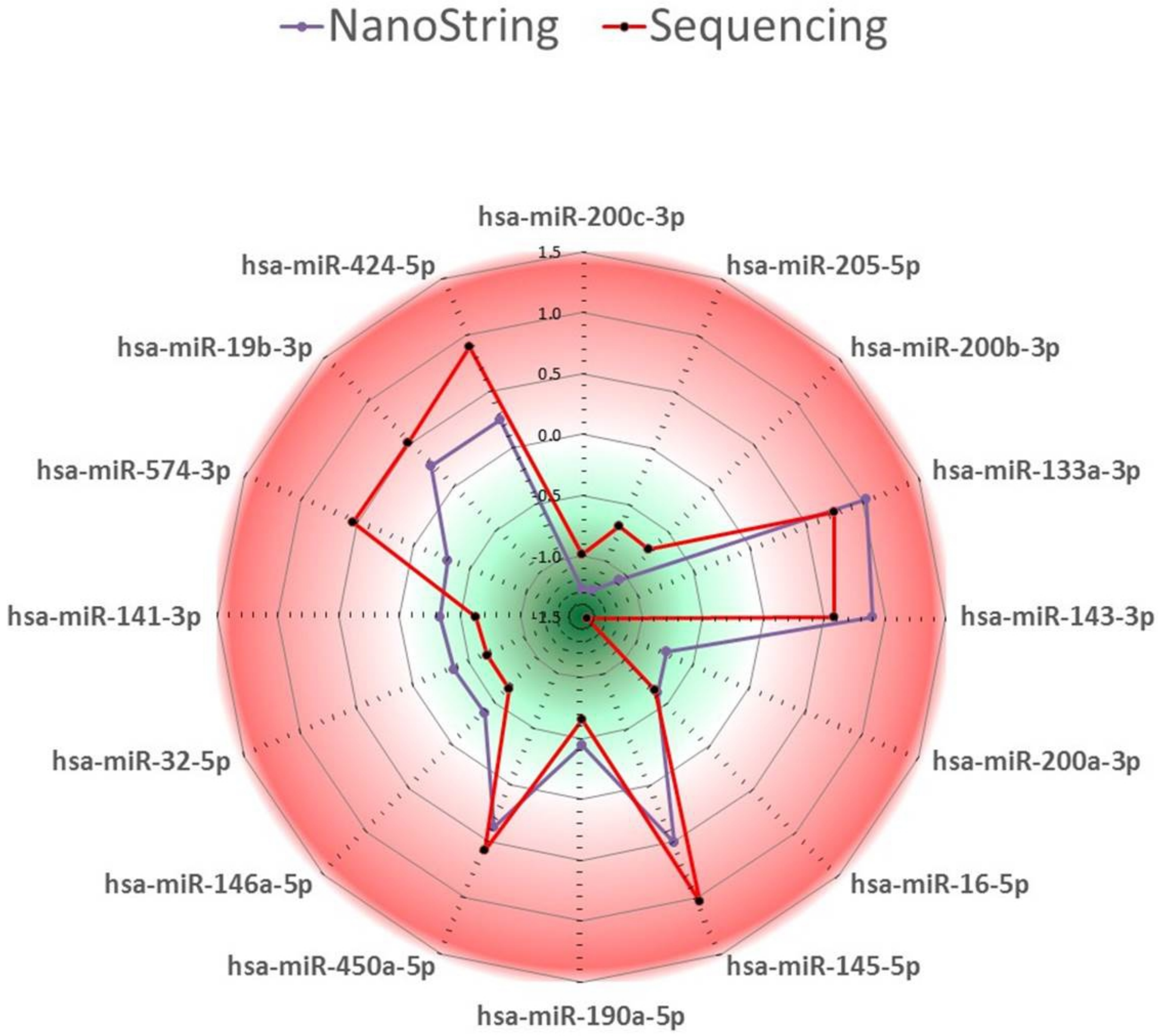
NanoString
Sequencing
hsa-miR-200c-3p
hsa-miR-205-5p
hsa-miR-200b-3p
hsa-miR-133a-3p
hsa-miR-143-3p
hsa-miR-200a-3p
hsa-miR-16-5p
hsa-miR-145-5p
hsa-miR-190a-5p
hsa-miR-450a-5p
hsa-miR-146a-5p
hsa-miR-32-5p
hsa-miR-141-3p
hsa-miR-574-3p
hsa-miR-19b-3p
hsa-miR-424-5p
1.5
1.0
0.5
0.0
-0.5
-1.0
-1.5

Fig. S3
